# Home blood pressure monitoring: a position statement from the Korean Society of Hypertension Home Blood Pressure Forum

**DOI:** 10.1186/s40885-022-00218-1

**Published:** 2022-10-01

**Authors:** Sang-Hyun Ihm, Jae-Hyeong Park, Jang Young Kim, Ju-Han Kim, Kwang-Il Kim, Eun Mi Lee, Hae-Young Lee, Sungha Park, Jinho Shin, Cheol-Ho Kim

**Affiliations:** 1 Division of Cardiology, Department of Internal Medicine and Catholic Research Institute for Intractable Cardiovascular Disease, College of Medicine, The Catholic University of Korea, Seoul, Republic of Korea; 2 Department of Cardiology in Internal Medicine, Chungnam National University Hospital, Chungnam National University College of Medicine, Daejeon, Republic of Korea; 3 Division of Cardiology, Department of Internal Medicine, Yonsei University Wonju College of Medicine, Wonju, Republic of Korea; 4 Department of Cardiology, Chonnam National University Hospital, Gwangju, Republic of Korea; 5 Division of Geriatrics, Department of Internal Medicine, Seoul National University Bundang Hospital, Seongnam, Republic of Korea; 6 Division of Cardiology, Department of Internal Medicine, Wonkwang University Sanbon Hospital, Gunpo, Republic of Korea; 7 Department of Internal Medicine, Seoul National University, Seoul, Republic of Korea; 8 Division of Cardiology, Severance Cardiovascular Hospital and Integrated Research Center for Cerebrovascular and Cardiovascular Diseases, Yonsei University College of Medicine, Seoul, Republic of Korea; 9 Division of Cardiology, Department of Internal Medicine, Hanyang University College of Medicine, Seoul, Republic of Korea

**Keywords:** Blood pressure, Ambulatory blood pressure monitoring, Hypertension, Measurement, Prevention and control, Home blood pressure

## Abstract

Home blood pressure measurement (HBPM) has the advantage of measuring blood pressure (BP) multiple times over a long period. HBPM effectively diagnoses stress-induced transient BP elevations (i.e., white coat hypertension), insufficient BP control throughout the day (i.e., masked hypertension), and even BP variability. In most cases, HBPM may increase self-awareness of BP, increasing the compliance of treatment. Cumulative evidence has reported better improved predictive values of HBPM in cardiovascular morbidity and mortality than office BP monitoring. In this position paper, the Korean Society of Hypertension Home Blood Pressure Forum provides comprehensive information and clinical importance on HBPM.

## Introduction

Home blood pressure monitoring (HBPM) has three characteristics: (1) monitoring is performed by the patients themselves; (2) measurements are performed at the home; (3) an automatic blood pressure (BP) device is used rather than a conventional manometer. These traits result in several advantages and limitations.

First, self-monitoring means that healthcare professionals do not take part in the measurement. BP may be elevated due to conscious or unconscious anxiety in the presence of healthcare providers. This phenomenon is known as the white coat effect, which is the reason for diagnosing hypertension in some persons and leading to unnecessary medication. The white coat effect can be avoided if the patients measure their own BP. During this process, the patient should take the initiative, and this includes purchasing a device to self-monitor. This may eventually improve disease awareness and treatment compliance. However, a correct understanding of the monitoring method and continuous training require non-healthcare professionals to self-monitor their BP. Since most people consider purchasing a BP manometer only after being advised by a physician, it is necessary for physicians to firstly recognize the importance of HBPM. In addition, if reliable education on HBPM is not provided to healthcare professionals and patients, reliable BP data may not be obtained.

Second, monitoring BP at home has the advantage of obtaining accurate BP measurements while avoiding the anxiety that patients may experience when they visit a medical facility. Also, mean BP and trends in BP fluctuation can be obtained with repeated monitoring. However, whether the BP at rest represents a person's overall risk of cardiovascular events remains debatable. The disadvantage of only using only BP monitored at rest is that it is impossible to obtain high BP measurements during work or outdoor activities involving exercise or stress. As a result of these challenges, HBPM may not be an optimal alternative.

Lastly, there are advantages and disadvantages associated with using an automatic BP manometer. Most automatic BP manometers use oscillometric measurements to measure diastolic BP using a constant formula that considers the pressure where oscillations begin and where they have maximum amplitude. Therefore, concerns have been raised regarding accuracy as a single BP formula cannot function accurately for every patient.

This statement for HBPM were prepared to provide comprehensive information on HBPM, considering the above benefits and limitations. A survey of 330 physicians on HBPM was conducted in 2016. In the survey, 89.4% of the respondents said that HBPM is necessary or very necessary, but 30% of the respondents said that HBPM is more important than clinic BP monitoring. Meanwhile, 49.7% of the respondents said that they recommend HBPM to patients, thereby showing that it is necessary to improve physicians’ thinking in the role and usefulness of HBPM.

The purpose of this statement is to share more professional knowledge about HBPM. In addition, we hope to avoid the white coat effect, and will be able to identify more patients with masked hypertension.

### Previous guidelines for home blood pressure monitoring

Since BP monitoring using a mercury BP manometer has been available, BP levels measured at home are usually lower than those measured in an office setting. However, auscultatory measurement using a mercury-based BP manometer at home is not widely used because it is difficult to perform and requires professional training. In 1981, Donald Nunn invented a completely automatic BP manometer, making ambulatory BP monitoring (ABPM) and HBPM possible. Following this invention, many studies highlighting the importance of ambulatory BP have been conducted in Europe. In Japan, it was reported that HBPM is not less significant compared to ABPM. Active research has led to the emergence of a field called chronologic medicine regarding BP changes, hormonal changes, and differences in occurrence of cardiovascular diseases in a day. The concept of morning hypertension, nocturnal hypertension, and nighttime dipper/nondipper were introduced and their clinical significance was supported by the ABPM study results.

Nocturnal hypertension or nighttime nondipper status has been linked to an increased risk of cardiovascular events. Although short-term BP variability throughout the day can be assessed using ABPM, variability assessment is not feasible with HBPM. This is cited as a limitation of HBPM; however, the clinical significance of short-term BP variability is uncertain. Several studies have monitored home BP and observed its relationship to cardiovascular events. These studies have shown that patients with white coat hypertension have higher future hypertension and cardiovascular events than people with normal BP.

Japan has had many research achievements related to home BP. In 2003, a guideline on HBPM was published. A comparable guideline was published in Korea by the working group for BP monitoring of the Korean Society of Hypertension (KSH) in 2007, specifically as a chapter of the Guideline of Self-Measuring of BP. However, the 2007 version lacked expertise because the contents were too simple and only covered recommendations on monitoring methods and their effectiveness without any background explanation. Professor Imai's second guideline on HBPM was published in Japan in 2012. The clinical significance of HBPM, selection of a BP monitor, reliability of BP monitors, monitoring method, and recording and archiving of monitoring data is described; however, there were no substantial changes compared to the previously published guideline.

In Europe, a guideline on home BP was published in 2000 and revised in 2008. Europe has focused on ABPM and did not take much initiative in recommending HBPM, possibly because the majority of home BP monitors have been produced by companies of Asian countries, including Japan and Taiwan, home BP devices have become widely available and affordable, but ABPM still requires expensive machinery and testing fees. Over time, the importance of home BP has increased in Europe as studies have reported that the association between home BP and cardiovascular events is not weaker than the association between ambulatory BP and cardiovascular events. With these findings, the 2008 guideline recommended HBPM more strongly. However, no further guideline has been published since 2008, and the existing content of the guideline is covered in the Guideline on Hypertension published by the European Society of Hypertension.

### Clinical significance of home blood pressure monitoring

HBPM enables the assessment/prediction of risk for hypertension-mediated target organ damage (TOD) and cardiovascular events. Hypertension-mediated TOD can indicate poor BP control, as summarized in Table 1. The presence of TOD indicates that the target BP is not being achieved [1]. The incidence of left ventricular hypertrophy (LVH), as identified by electrocardiogram, was lower with strictly controlled home BP was compared with routine home BP control. Furthermore, elevated home BP is more strongly associated with an increase in LV muscle mass, and the presence of LVH compared to elevated office BP [2, 3]. In particular, morning home BP is more closely associated with LVH compared to office BP or evening home BP [2]. Proteinuria and carotid artery intima-media thickness (IMT) is more closely correlated with home BP than office BP [4, 5], and the carotid artery IMT is more closely correlated with systolic home BP than systolic office BP (0.34 vs. 0.25, *P* < 0.001) [4]. While urinary albumin excretion rate is correlated with diastolic office BP (correlation coefficient, 0.31; *P* < 0.05), home BP is correlated with both systolic (correlation coefficient, 0.28; *P* < 0.05) and diastolic BPs (correlation coefficient, 0.26; *P* < 0.05) [5].

**Table 1:** Target organ damage

Organ	Target organ damage
Brain	Stroke, ischemic or hemorrhagic
	Transient ischemic attack
	Cognitive impairment
Eye	Hypertensive retinopathy
Heart	Left ventricular hypertrophy
	Nonvalvular atrial fibrillation
	Heart failure, reduced ejection fraction or preserved ejection fraction
Kidney	Chronic kidney disease
	Proteinuria/albuminuria
Blood vessel	Increased aortic stiffness ^a)^
	Aortic aneurysm
	Carotid artery atherosclerosis
	Peripheral artery occlusive disease ^b)^

^a)^ Brachial-ankle pulse wave velocity ≥ 18 m/sec

^b)^ Ankle-brachial index < 0.9

Home BP is critical in predicting the risk of cardiovascular events. According to a prospective study, home BP predicts the incidence of cardiovascular events more accurately than office BP [6–9]. The superiority of home BP over office BP in prognosis monitoring was first shown in the Ohasama study in Japan, which included 1,789 subjects. In early BP monitoring, repeated systolic home BP had a stronger predictive power for total mortality (hazard ratio [HR], 1.014; 95% confidence interval [CI], 1.003–1.025; *P* < 0.05) and cardiovascular death (HR, 1.021; 95% CI, 1.001–1.041; *P* < 0.05) compared to office BP monitoring in clinics [9]. In the Pressioni Arteriose Monitorate E Loro Associazioni (PAMELA) study in 2,051 Italian subjects, the risk of death increased more with a given increase in home BP than office BP [10]. Finally, in the Finn-Home study, which enrolled 2,081 subjects in Finland, systolic and diastolic home BP had a stronger predictive power compared to office BP in fatal and nonfatal cardiovascular events (systolic home BP: HR 1.23, 95% CI 1.13–1.34, *P* < 0.01; diastolic home BP: HR 1.18, 95% CI 1.10–1.27, *P* < 0.01). However, only systolic home BP was a significant predictor of mortality (systolic home BP: HR 1.11, 95% CI 1.01–1.23, *P* = 0.04), while the office BP was a significant predictor of mortality [8].

Different methods of monitoring BP may provide information complementing each other. Also, discordance between the office BP and out-of-office BP may help detect white coat hypertension and masked hypertension. Patients with these forms of hypertension tend to, over the long-term, have worse organ outcomes than people with normal BP. Patients with masked hypertension (office BP < 140/90 mmHg and home BP ≥ 135/85 mmHg) show a markedly higher risk of cardiovascular events [11]. Although with 24-h ambulatory BP, nocturnal BP is not possible with HBPM, its reproducibility is comparable to that of ABPM [12]. As ABPM requires devices from a healthcare facility, its shortcoming lies in that it can only be performed for a limited amount of time. In contrast, HBPM allows for regular long-term BP monitoring. BP variability may be assessed if BP is monitored at designated times under standardized conditions. In addition, home BP is more closely associated with hypertension-mediated organ damage and incidence of cardiovascular events [13, 14]. Although ABPM is the ideal option [10, 15] a number of studies and meta-analyses have shown that HBPM is comparable to ABPM in detecting asymptomatic TOD [5, 16, 17].

Thanks to recent remarkable technical advancements, it has become possible to measure nocturnal BP at home using a preprogrammed automated device [18]. Nocturnal home BP is closely related to nocturnal ambulatory BP. According to a meta-analysis, the difference between nocturnal home BP measurements and nocturnal ambulatory BP measurements is 1.4 mmHg (95% CI, 0.3–2.6 mmHg) for systolic BP and –0.2 mmHg (95% CI, –0.9 to 0.6 mmHg) for diastolic BP [19]. Although studied in a limited number of patients, nocturnal home BP was associated with LV mass index, albuminuria, and hypertension-mediated TOD, including carotid artery IMT [19]. In the Japan Morning Surge-Home Blood Pressure (J-HOP) study conducted in 2,545 Japanese subjects, nocturnal home BP was an independent predictive factor for carotid artery disease and stroke regardless of morning home or office BP monitored [20]. In this study, nocturnal home BP taken at 2, 3, and 4 in the morning were significant predictive factors for cardiovascular events (total cardiovascular events: HR 1.177, 95% CI 1.021–1.356, *P* < 0.05; stroke: HR 1.236, 95% CI 1.006–1.520, *P* < 0.05).

In conclusion, despite a number of limitations, HBPM is superior for predicting hypertension-mediated TOD and risk of cardiovascular events compared to office BP and comparable with 24-h ambulatory BP. Furthermore, as recent technical developments have made the nocturnal HBPM possible, using these together may improve outcomes of BP management.

### Principles of blood pressure monitoring

#### General methods of blood pressure monitoring

In general, the methods by which BP is determined are categorized as follows: first, the oscillometric method uses the rhythm or the extent and shape of oscillations obtained by compressing the blood vessels to measure BP; second, the auscultatory method measures the BP by listening with a stethoscope for sounds produced while compressing a blood vessel. However, the auscultatory method is not feasible for the general public in settings other than healthcare facilities. Unless a device for HBPM equipped with sound analysis artificial intelligence is developed in the future, the HBPM may be limited to employing the oscillometric method.

An oscillometric BP monitor is a non-mercury BP manometer categorized as an automatic electric BP manometer and is. It is commonly referred to as an automated device because reading can be obtained by simply clicking a button. Recently, this has been improved to automated BP monitoring, which follows a predefined protocol, such as "after a 5-min rest, perform three measurements with a 1-min break," and indicates the result immediately. However, most HBPM devices use an automated device that measures BP once and displays the result with one click. Therefore, oscillometric automated devices are conveniently used for HBPM.

#### Principles behind oscillometric automated devices

It is recommended to use a BP manometer validated according to the international verification protocol for oscillometric automated devices. However, manufacturers do not disclose the specific details of the algorithms used by individual devices because of intellectual property issues. If the algorithms were disclosed, this could help patients at high risk of measurement errors avoid such devices [21].

#### The pressure at maximum oscillation is the mean arterial pressure

**Fig. 1:**
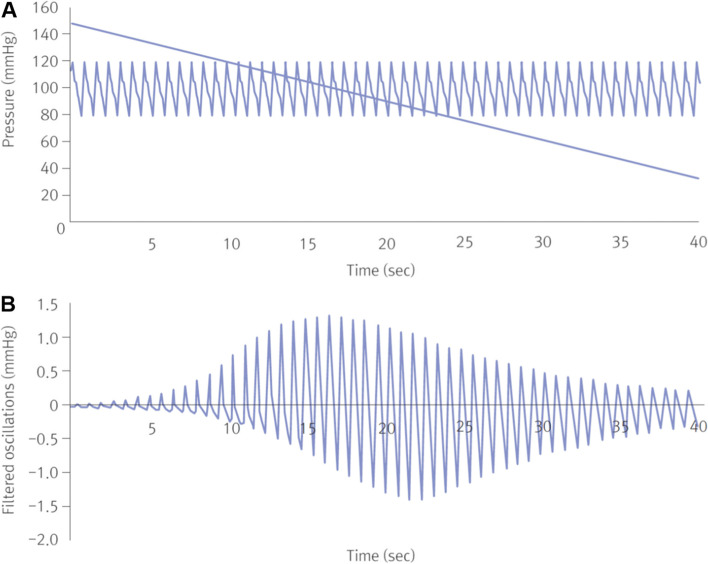
**A** Fluctuation phase of blood pressure (BP) by change of compression band pressure. **B** The oscillation due to change in compression band pressure observed by enlarging the slope in (**A**). Reprinted from Babbs et al. [22]

During the measurement process, the air is infused into a rubber bladder located in a nonelastic cuff wrapped around the upper arm until blood flow to the arteries in the upper arm is completely blocked. The pressure inside the rubber bladder at this point is defined as the cuff pressure. The blood flow is blocked when the cuff pressure surpasses the systolic pressure, and once it is lower than the systolic pressure, blood flows again with pulsations following the cardiac cycle. In other words, the cuff pressure acts as a high-pass filter. When the cuff pressure is higher than the systolic pressure, blood vessel volume increases in response to the cardiac cycle, which applies pressure to the cuff, decreasing the cuff volume and increasing cuff pressure correspondingly by Boyle's law. In addition, as blood vessel volume decreases, cuff pressure decreases, causing oscillations. One oscillation occurs over one cardiac cycle. If the cuff pressure is lower than the systolic pressure, a larger oscillation occurs due to the volume change of a number of blood vessels. The mean arterial BP is the pressure when the largest oscillation is observed as the cuff pressure gradually decreases from maximum to minimum (Fig. 1) [22, 23].

#### Oscillation ratio or systolic/diastolic pressure detection ratio

**Fig. 2:**
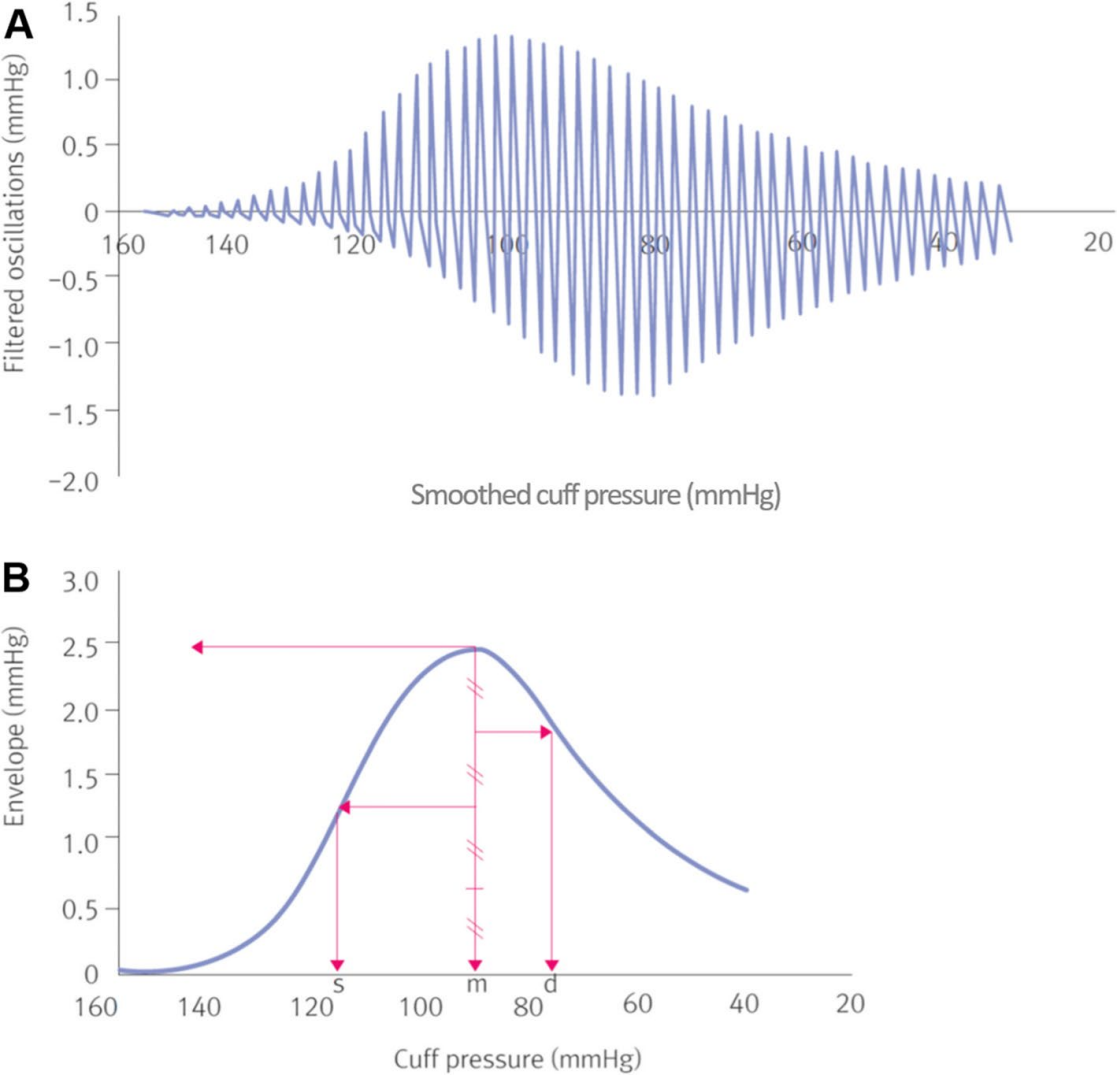
**A** Oscillation by compression band pressure. **B** Defined the pressure at maximum oscillation amplitude mean pressure (m) and the pressure in the escalating phase at 50% of maximum amplitude, systolic pressure (s), and the compression band pressure in the declining phase at 75% of maximum amplitude, diastolic pressure (d)

The period when the compression band pressure is higher than the pressure at maximum oscillation is called the escalating phase, and when it is lower, the declining phase. Systolic pressure is the BP at which the amplitude of oscillations observed is 50% of the maximum oscillation during the escalating phase. The diastolic pressure is the BP at which the amplitude of oscillations observed is 75% of the maximum oscillation during the declining phase. The oscillation ratio is an essential algorithm obtained empirically in oscillometric BP monitoring. This empirical ratio is sensitively affected by pulse pressure and aortic stiffness (Fig. 2).

Measuring BP using this fundamental principle has its shortcomings. For example, from a physiological point of view, a comprehensive model should include the concept of dynamic compliance. In addition, the effect of the so-called transluminal pressure applied directly to the artery due to the difference between the pressure of the compression band artery must be considered. A comprehensive simulation that takes all of these factors into account shows a significant difference depending on the dynamic compliance and spasticity of the artery. This means that even in an oscillometric automated device with an optimized algorithm, there can be significant variation in accuracy between individuals. Furthermore, it is difficult to obtain a stable BP value in patients with high BP variability unless BP monitoring is repeated a sufficient number of times.

### Mechanism of auscultatory automated devices

In the early 1990s, auscultatory automatic BP devices had already passed the British Hypertension Society (BHS) validation standards [24]. These devices were validated for ABPM; however, they were not employed in Korea. In theory, this method is the closest to the auscultatory manual BP monitoring method because it uses a microphone to monitor BP by directly detecting K1 and K5 instead of calculating Korotkoff sounds. However, since the process for detecting mechanical sound waves may differ from that of the human body, there may be several methods available to set the thresholds for the frequency and intensity of sound waves that the human body can recognize. In addition, hardware performance has improved significantly in recent years compared to the 1990s. As new methods, such as those using machine learning, have emerged, auscultation-type automated devices are on the verge of development and will soon be available.

In conclusion, oscillometric automated devices operate by deriving systolic and diastolic pressure from mean BP using the maximum oscillation amplitude, oscillation ratio, and several standard indicators. However, the specific algorithms used by devices have not been disclosed. Therefore, the accuracy of a device in actual clinical practice should be evaluated according to the international oscillometric automated device validation protocol. In particular, certain groups, such as the elderly, pregnant women, pediatric patients, patients with a very thin or thick upper-arm circumference, and patients with arterial stiffness, who were highly likely not to have been taken into account when the oscillometric BP manometer algorithms were designed, should be cautious of measurement errors. Auscultatory ABPM was partially limited to ABPM, but an auscultatory automated device that can be used in the clinic or home setting will be developed as hardware further improves.

### Blood pressure measurement devices

#### Blood pressure measurement devices

Home BP is monitored using oscillometric automated devices. Auscultatory or semi-automatic BP devices are not recommended. There are three types of devices available based on measurement location: finger, wrist, and upper arm.

#### Finger devices

Devices that measure BP using a finger are not recommended because of their inaccuracies due to measurement distortion with peripheral vasoconstriction, BP alteration as the finger is too far away from the heart, and the effect of limb position on BP.

#### Wrist devices

Devices that measure BP at the wrist have the same last two problems as the finger devices. Inaccurate measurements may be obtained if the wrist device is not placed at the heart level or if the wrist is flexion or hyperextension during measurement. Also, if the radial artery is not fully compressed, both the radial and ulnar artery oscillation waves influence BP values, making it difficult to produce an accurate algorithm to estimate systolic and diastolic BP and, ultimately, they lead to obtaining inaccurate BP values. However, wrist devices may be considered in special populations, such as obese or elderly individuals, in whom the upper arm is more challenging to perform BP measurement. Because the correct placement of the cuff at heart level is essential for an accurate measurement, some wrist devices have built-in sensors to indicate the correct position. Wrist devices are commonly used among patients despite their limitations because the measurement is readily performed without removing the clothes. Approximately 20 wrist devices that passed validation protocol with established protocols are currently available in the market.

#### Upper arm devices

Devices that measure BP in the upper arm are the most reliable in both clinical practice and major hypertension trials for HBPM. Most international recommendations for oscillometric automated office BP measurement devices also apply to automated home BP devices. For accurate BP measurement, it is important to use validated home BP devices according to established protocols and to select a cuff to fit the upper-arm circumference. Many new HBPM devices have a built-in memory that automatically stores BP readings. Commonly, home BP devices are inappropriate in patients with arrhythmias. Recently, some devices can detect BP during irregular heart rhythm, such as atrial fibrillation. The majority of HBPM devices assess daytime BP, but some devices become available to measure during sleep thus they can detect sleep BP. Also, some devices are available to transmit BP data to the patients and their healthcare providers via Bluetooth. Approximately 200 devices that passed the validation protocol with established protocols are available on the market.

### Cuffs

In an auscultatory BP sphygmomanometer, the role of the BP cuff is to compress the artery with the inflatable part of the BP cuff (bladder), whereas, in an oscillometric automated device, the cuff is at the signal sensor of oscillation of the artery. Thus, it is also important to document the cuff's type, shape, and material characteristics under the device validation procedure. The cuff size is selected by upper arm circumferences according to the device’s instructions (Table 2). Furthermore, cuff size influences the accuracy of the BP measurement, while the upper arm circumference and the shape of the arm might be considered when choosing a cuff for obese patients. However, its relevant recommendation is not currently available. Some models have markers to indicate the proper position or acceptable range of cuff. D-ring cuffs are generally used as self-application cuffs.

**Table 2:** Standard cuff size by arm circumference (ACC/AHA recommendations)

Classification	Upper arm circumference (cm)	Bladder dimension (width × length, cm)
Small adult	22–26	12 × 22
Adult	27–34	16 × 30
Large adult	35–44	16 × 36
Extra-large adult	45–52	16 × 42

The size of bladder and cuff varies by manufacturer

*ACC/AHC* American College of Cardiology/American Heart Association

### Device selection

A proper device should be selected to measure BP accurately (Table 3).

**Table 3:** Proper device and cuff selection

Considerations
1. Use oscillometric automated device that has been validated with established protocols
2. Use upper-arm cuff device
3. Finger device is not recommended, and wrist device is not inaccurate if it is not placed at heart level
4. Select a cuff to fit patient`s arm circumference
5. Options ･Consider the simplest devices to push a button to initiate a reading, and then to be displayed on the screen after the reading is taken ･Consider the programmable device to take multiple readings with option to set the time interval between readings ･Consider the device to be stored readings automatically, to be printed and to be transmitted the blood pressure (BP) readings to health providers ･Consider the device to assess the presence of the irregular beats (atrial fibrillation)
6. Measure BP in both arms initially, and then the arm with the higher BP is used at subsequent measurement

### Validation of blood pressure monitors

For the accurate diagnosis of hypertension, the BP device is validated according to established protocols. However, unfortunately, less than 20% of devices on the market have been validated. Validation of BP devices began in ad hoc protocols in the 1980s, and then reputable organizations published validation procedures. The US Association for the Advancement of Medical Instrumentation (AAMI) and the BHS published more systemized standard protocol for clinical validation of automated BP devices against a mercury sphygmomanometer as a reference value. In 2002, the European Society of Hypertension (ESH) Working group developed the ESH-International Protocol (ESH-IP), and it was stringently revised in 2010. ESH-IP 2002 was widely used and more practical because the sample size and pass criteria were simplified compared to previous protocols. In 2018, the universal protocol (Association for the AAMI/ESH/Organization for Standardization [ISO]), single protocols for global acceptance, was developed. This protocol recommends that reference BP measurements for validation can be obtained using mercury sphygmomanometers or non-mercury sphygmomanometers (aneroid or other) that fulfill the ISO 81060–1 requirement for accuracy (maximum permissible error shall be ± 1 mmHg) because of concern with mercury toxicity in many countries (Table 4). AAMI, ESH-IP, BHS, and ISO have provided a list of validated BP devices and pass criteria of their respective protocols at http://www.dableducational.org, and a list of internationally validated BP devices can be found on the KSH website.

**Table 4:** Universal protocol of validation

Parameter	Recommendation
Efficacy measure	Threshold for BP measurement accuracy acceptance at estimated probability of tolerable error (≤ 10 mmHg, ≥ 85%)
Sample size and age	General population: ≥ 85 participants, aged > 12 years
Sex	≥ 30% male and ≥ 30% female
Reference BP	Mercury sphygmomanometers or non-mercury sphygmomanometers those fulfill requirement for accuracy (maximum permissible error shall be ± 1 mmHg)
Test requirements (number)	Each of reference and test device ≥ 255
BP ranges	SBP: ≤ 100 (≥ 5%), ≥ 160 (≥ 5%), ≥ 140 mmHg (≥ 20%) DBP: ≥ 60 (≥ 5%), ≥ 100 (≥ 5%), ≥ 85 mmHg (≥ 20%)
Cuff size	• Cuff size-stratified subgroups with ≥ 2 cuffs → minimum of 22 subjects per cuff •Set for the distribution of the participants arm circumference according to the specified range of use of the test device
Special populations	• Special populations: age < 3 years /pregnancy / arm circumference > 42 cm/atrial fibrillation, and others • Special population studies include ≥ 35 participants after successful general population study • Pregnancy: 45 subjects, Korotkoff K5 for reference DBP • Children: 35 subjects, aged 3–12 years analyzed together with ≥ 50 years older subjects. Korotkoff K5 for reference DBP
Data collection	Same-arm sequential BP measurement
Pass criteria	• Criteria 1 (BP value): average BP difference ≤ 5 mmHg, standard error ≤ 8 mmHg • Criteria 2: tolerable error • Absolute BP differences ≤ 5, 10, 15 mmHg • Scatterplots (standardized Bland–Altman plot)

*BP* blood pressure, *DBP* diastolic BP, *SBP* systolic BP

In conclusion, it is essential to use validated devices according to established protocols with a proper cuff to fit arm upper-arm circumference for accurate home BP measurement. Recently, devices have been available to assess atrial fibrillation to improve device accuracy on the market. A list of validated BP devices monitors can be reference assessed at http://www.dableducational.org/ and the universal protocol for device validation is currently used for the device validation [25–32].

### Home blood pressure monitoring method

BP measurements may fluctuate due to various factors such as respiration, stress, medication, environment, and circadian rhythms. Therefore, diagnosing and treating hypertension based on only one or two clinic BP measurements may result in an inappropriate diagnosis and unnecessary treatment. Due to this limitation of clinic BP-based management, the use of ABPM is increasing. The advantage of ABPM is that the BP is presented as a mean value of multiple measurements of BP done in 15-min to 30-min intervals. This more accurately reflects actual BP and the pressure load experienced by patients over 24 h. Therefore, ambulatory BP is more relevant than clinic BP in estimating the TOD and cardiovascular mortality. However, the most significant limitation of ambulatory BP is the difficulty of usage in a primary care setting. As an alternative, home BP can relatevely accurately monitor the patient's mean BP. Its relevance concerning cardiovascular disease compared to clinic BP is supported by the Ohasama cohort study, Finn-Home study, and HONEST study [8, 9, 28, 33] However, in order for it to be used in clinical settings, it must be (1) monitored using a standard approach, and (2) measured with a validated BP manometer. The validated home BP manometers are mostly oscillometric devices and can be referenced at http://www.dableducational.org or http://bhsoc.org/bp-monitors/bp-monitors/for-home-use.

As mentioned above, if BP is monitored at home using a standardized approach, its predictive ability for cardiovascular events may be superior to clinic BP. Also, the Japanese Society of Hypertension (JSH) recommends home BP over 24-h ambulatory BP for out-of-office BP monitoring.

For accurate HBPM, the following must be considered: (1) one must refrain from caffeine consumption, exercise, smoking, bathing, and alcohol consumption 30 min prior to monitoring; (2) morning BP must be monitored after using the toilet within 1 h of waking up in the morning, before eating breakfast, and before taking morning antihypertensive drugs; (3) nocturnal BP must be monitored within 1 h of going to bed; (4) each time, BP should be measured twice 1–2 min apart in a seated position after resting for 1–2 min; (5) the cuff should be wrapped around a bare arm if possible but it can be placed over clothes if the patient is wearing thin clothes; and (6) BP should be monitored for at least 1 week at initial diagnosis and at least 5–7 days prior to the outpatient visit [34–36]. The Ohasama cohort study was performed on 1,500 Japanese subjects who were ≥ 40 years old without a history of stroke and suggests that frequent long-term BP monitoring is favorable. In subjects who monitored home BP an average of 25 times, the risk of stroke increased by 35% for every 10 mmHg increase in systolic BP and in subjects who monitored home BP for a total of 2 days, the risk of stroke increased by 20% for every 10 mmHg increase in systolic pressure, thereby suggesting that stroke predicting ability improves with increasing frequency of home BP measurements [9]. Thus, frequent and long-term BP monitoring may enhance predictive ability. If circumstances do not allow that, HBPM for at least 5–7 days before a clinic visit is considered clinically useful. Although the resting time required prior to measurement varies by clinical practice, the ESH recommends a resting time of 5 min prior to taking a measurement. However, because this may reduce compliance, the JSH and KSH recommend 1–2 min resting time [34–37].

If appropriate measurements are taken following a standardized protocol and the mean BP is ≥ 135/85 mmHg, a diagnosis of hypertension can be made. Also, if a patient is already being treated for hypertension, then they meet the definition of uncontrolled hypertension. The JSH recommends using home BP to determine whether a patient has genuine hypertension when clinic BP is ≥ 140/90 mmHg and using 24-h ambulatory BP if HBPM is not feasible [37]. Morning BP ≥ 135/85 mmHg with a mean BP of ≤ 135/85 mmHg is defined as morning hypertension. In addition, it has been reported that insufficient morning BP control, even when clinic BP is low, indicates a high risk of cardiovascular events. HBPM has the following advantages: (1) it helps evaluate masked hypertension, white coat hypertension, and resistant hypertension; (2) it enables the assessment of morning BP, which would go unnoticed with clinic BP monitoring; and (3) it may enhance BP response by improving medication compliance. Above all, it is reported that predicting cardiovascular events is higher with BP monitored at home than in the clinic. As such, the recently revised hypertension management guidelines of the United States, Europe, Japan, and Korea all endorse out-of-office BP monitoring, especially HBPM, for the diagnosis and treatment of patients with hypertension [38, 39].

### Indications for home blood pressure monitoring in real world clinical status

#### Indications for home blood pressure monitoring

**Fig. 3:**
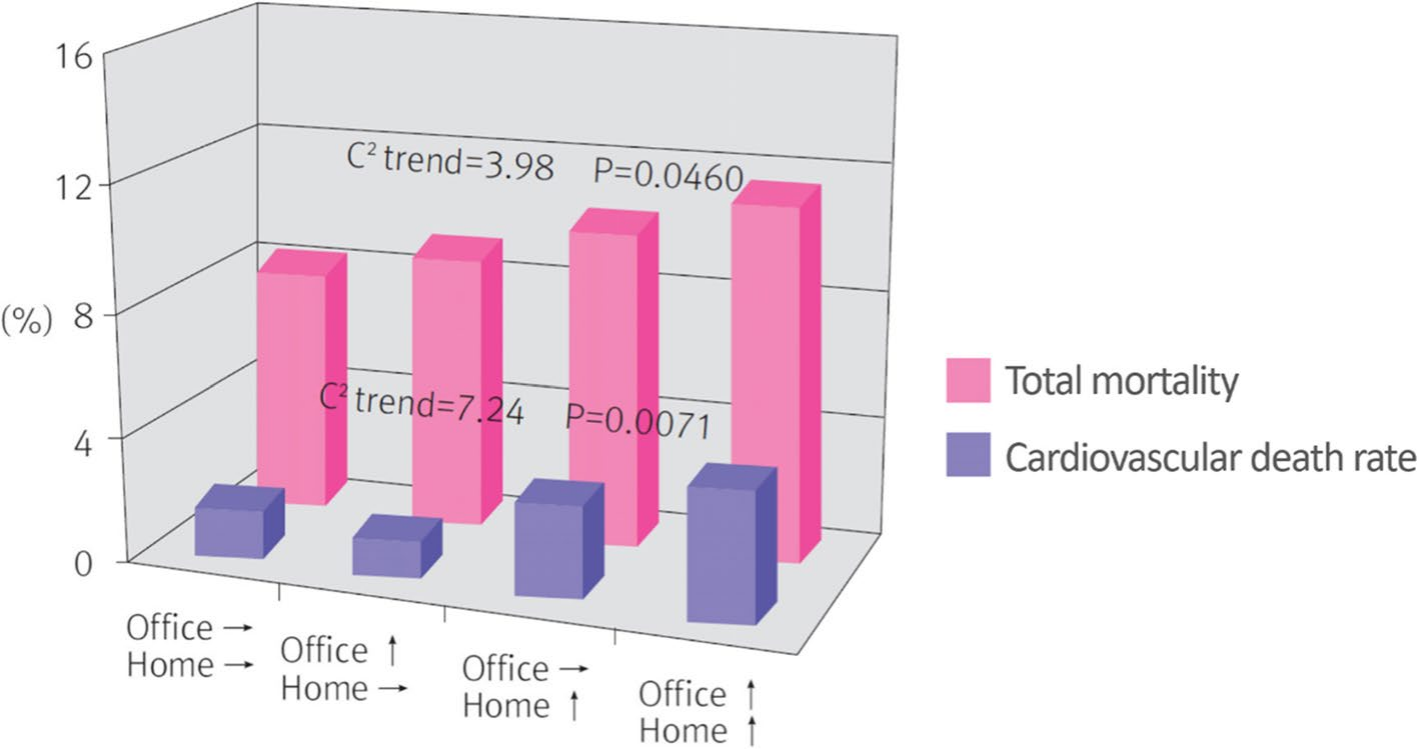
Trends in total mortality and cardiovascular death rate by office and home blood pressure. Reprinted from Mancia et al. [42], with permission of Wolters Kluwer Health

HBPM is supplemental to clinic BP monitoring and supported by the Global Hypertension Practice Guidelines of the International Society of Hypertension. Currently in Japan, Europe, and the United States, HBPM is recommended for daily monitoring of hypertension along with clinic BP monitoring [37, 40, 41]. In 2006, the Guideline on HBPM was published by the Korean Society of Home BP, and a new version was published in 2009 which recommends HBPM to physicians in local clinical settings. The 2018 Korean Guidelines for the Management of Hypertension also recommended HBPM (grade I recommendation, level of evidence A). It also recommended that all patients receive training on HBPM methodology to ensure accurate HBPM (grade I recommendation I, level of evidence C) [36]. The effectiveness of home BP was first proven in an epidemiological study among 1,789 residents aged ≥ 40 years in Ohasama, Japan for an average of 6.6 years. The study result showed a significant increase in total mortality and cardiovascular mortality associated with every 1 mmHg increase in home BP but not clinic BP [9]. In a study performed in Finland in approximately 2,000 patients, a 10 mmHg increase in BP measured in the clinic was associated with a relative risk of 1.01-for cardiovascular events, which was not statistically significant. Conversely, however, a 10 mmHg increase in home BP was associated with a relative risk of 1.22, thereby showing that home-measured BP is prognostically superior [8]. Compared with clinic BP, home BP more accurately reflects the incidence of cardiovascular disease and major organ damage in addition to mortality (Fig. 3) [42]. The benefits of home BP are that, unlike ambulatory BP, inter-organ monitoring is easy, monitoring is simple, convenience, and cheap. In addition, by involving patients directly in their hypertension management, treatment compliance is improved, the use of antihypertensive drugs is reduced, and appropriate treatment can be initiated based on an accurate diagnosis of hypertension. This, in turn, reduces mortality and the incidence of cardiovascular disease, thereby resulting in long-term economic benefits by reducing medical costs [43, 44].

#### Real world clinical status of home blood pressure monitoring

White coat hypertension, masked hypertension, morning hypertension and nocturnal BP cannot be detected by clinic BP monitoring. For these conditions, HBPM and ABPM are used to monitor BP outside the clinic. As home BP is generally lower than clinic BP, the diagnostic criteria for a hypertension diagnosis are based on home BP (BP ≥ 135/85 mmHg) [36].

#### White coat hypertension

**Fig. 4:**
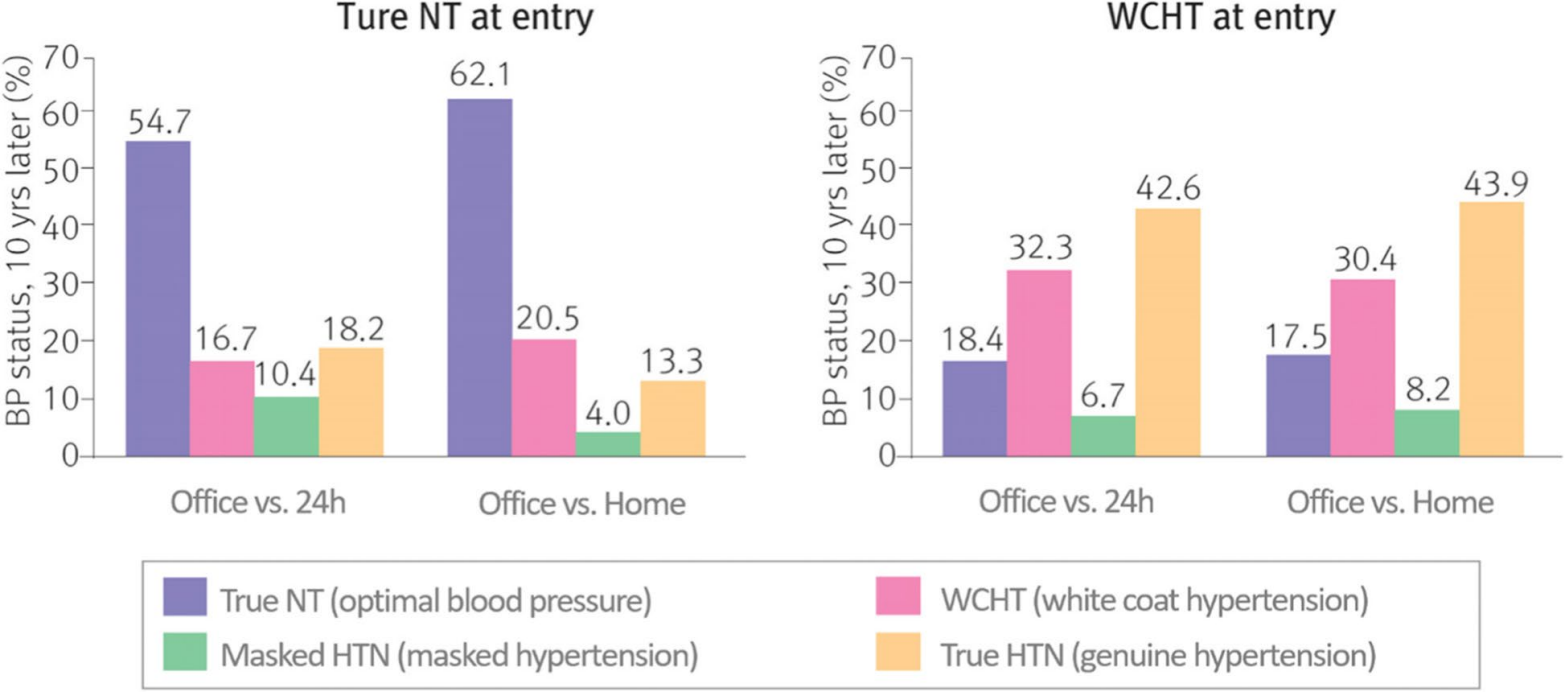
Transition from white coat hypertension to true hypertension. BP, blood pressure; NT, normotensive. Reprinted from Mancia et al. [49], with permission of Wolters Kluwer Health

The white coat effect refers to the difference between clinic BP and ambulatory BP or home BP. In other words, the measurements taken inside and outside the clinic may differ due to a transient BP elevation caused by the clinical or consultation setting. Therefore, white coat hypertension is defined as cases where the clinic BP is ≥ 140/90 mmHg while home BP or ambulatory BP is < 135/85 mmHg [36]. In the normal population, white coat hypertension may be suspected if BP is high despite the patient having fewer risk factors for hypertension with regard to their medical history, age, body weight, and smoking history. Although white coat hypertension is observed more frequently in females and the elderly, the contributing factors have not been identified yet. However, this occurs frequently when the sympathetic nervous system is easily activated due to tension [45, 46]. As an inaccurate diagnosis of hypertension may lead to adverse reactions due to unnecessary drug use, it is recommended to monitor home BP or ambulatory BP to exclude white coat hypertension if possible before medication is initiated [47]. Lifestyle adjustment is mainly recommended for patients with white coat hypertension, and periodic BP monitoring is recommended because there is a high risk of developing genuine hypertension in the future (Fig. 4) [48, 49].

**Fig. 5:**
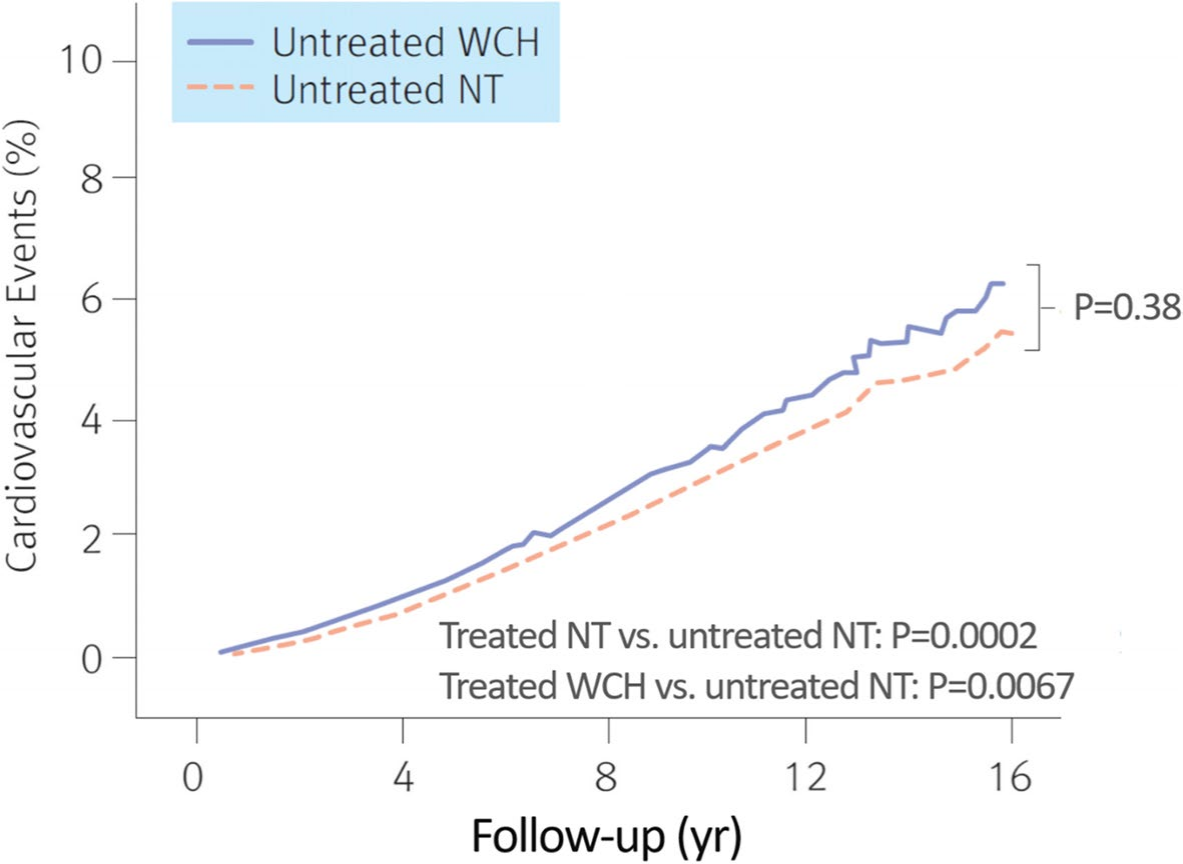
Incidence of cardio-cerebrovascular disease in patients with white coat hypertension patients and normal blood pressure. NT, normotensive; WCH, white coat hypertension. Reprinted from Franklin et al. [48], with permission of Wolters Kluwer Health

However, as there are reports that the morbidity rate of cardiovascular disease is higher in patients with white coat hypertension compared to people with normal BP, medication and lifestyle adjustment may be considered if white coat hypertension is accompanied by a metabolic disorder or asymptomatic organ damage (Fig. 5) [50].

#### Masked hypertension

**Fig. 6:**
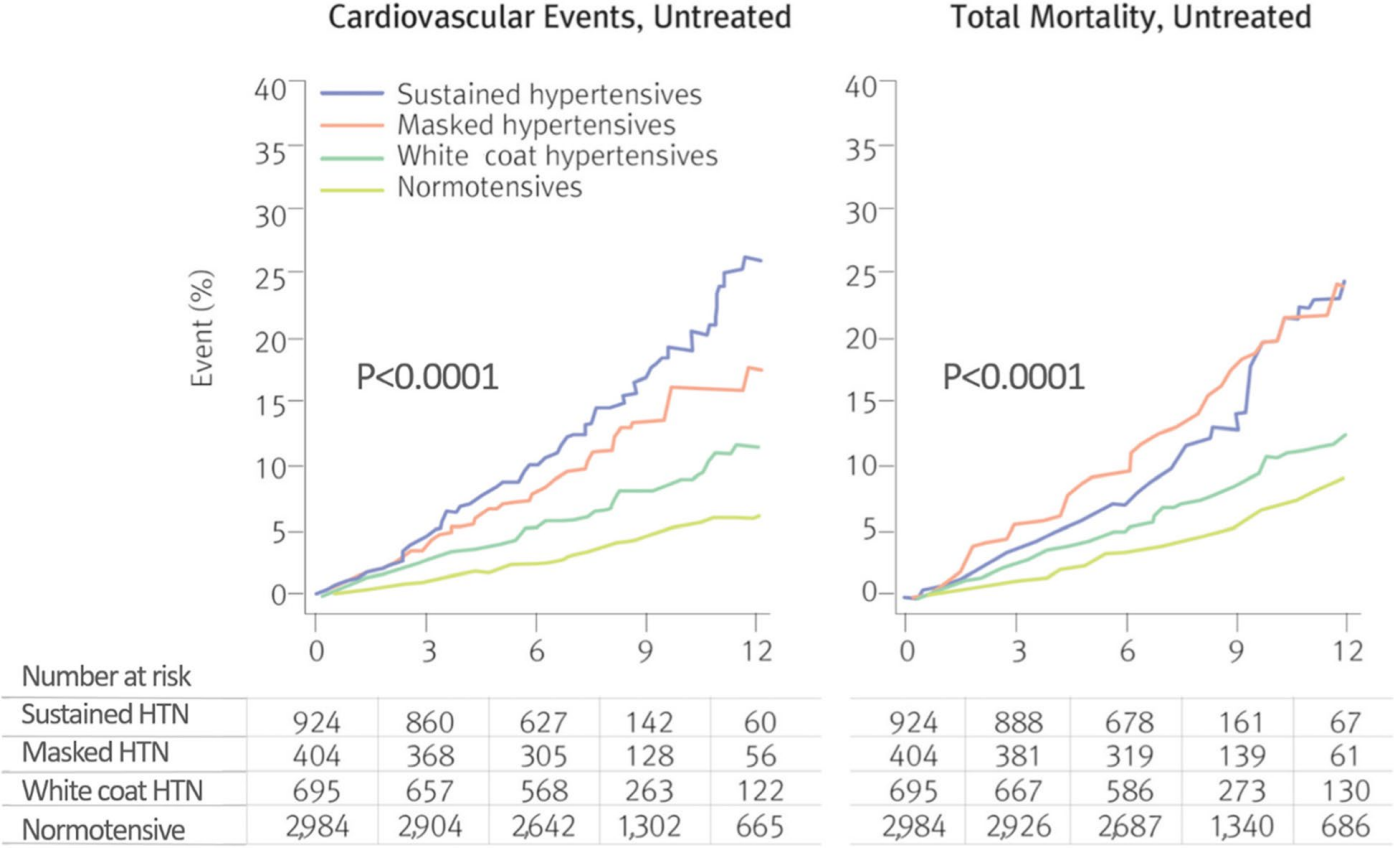
Cardiovascular events and total mortality according to the types of hypertension. HTN, hypertension; NT, normotensive. Reprinted from Stergiou et al. [53], with permission of Wolters Kluwer Health

Masked hypertension is defined as BP that is less than 140/90 mmHg when measured in the clinic while it is hypertensive when measured with HBPM or ABPM. The frequency of masked hypertension is 15%–30%. It is associated with factors such as male sex, smoking history, and alcohol consumption. It is also common in patients with high occupational stress, and those with a high BP variability are at a higher risk of masked hypertension [51, 52]. Since it has been reported that cardio-cerebrovascular risk is higher in masked hypertension compared to white coat hypertension, if masked hypertension is diagnosed, comprehensive medication should be considered (Fig. 6) [53]. It is recommended to test for masked hypertension and initiate medical treatment in patients at high risk of metabolic disorders, TOD, glycosuria, and chronic kidney disease (CKD), patients who smoke smokers and patients with high occupational stress [54–56].

#### White coat and mask effect on hypertension

White coat hypertension and masked hypertension are observed in the normal population that have never received antihypertensive treatment and in patients who are already on antihypertensive treatment. According to data from an ABPM registry, the percentage of patients taking antihypertensive medication exhibiting a white coat effect with high clinic BP but normal ambulatory BP (hypertension with white coat effect, white coat uncontrolled hypertension [WUCH]) was 13.5% of all treated patients. Also, 13.8% of patients on antihypertensive medication had uncontrolled hypertension misinterpreted due to the masked effect (hypertension with reverse white coat effect, masked uncontrolled hypertension [MUCH]) [36].

According to a cohort study performed in Spain involving approximately 60,000 patients, there was no significant increase in total mortality (HR, 1.06; 95% CI, 0.82 –1.37; P = 0.66) or cardiovascular mortality (HR, 1.04; 95% CI, 0.65–1.66; *P* = 0.86) in WUCH patients compared to patients with controlled hypertension [57]. However, in the meta-analysis conducted recently that included 12,000 MUCH patients, the risk of cardio-cerebrovascular disease and total mortality were increased compared to patients with controlled hypertension (HR, 1.80; 95% CI, 1.57–2.06) [58]. Studies on the prognosis of WUCH and MUCH are still lacking compared to those of white coat hypertension and masked hypertension; therefore, a large-scale comparative control study is needed [59].

### Diagnostic criteria and treatment from clinical guidelines

#### ESC/ESH guidelines for the management of hypertension

The ESH published the Practice Guidelines for Home Blood Pressure Monitoring 2014 and the 2018 ESC (European Society of Cardiology)/ESH Guidelines for the Management of Arterial Hypertension, which includes recommendations on HBPMs [39, 60] The guidelines recommend using a certified BP manometer for BP monitoring at home for 6–7 consecutive days or at least for three days before visiting a clinic. Monitoring should be completed in the morning and evening every day, and at least two measurements should be taken at 1 or 2 min intervals.

Based on home BP, the diagnostic criteria for hypertension is a BP ≥ 135/85 mmHg (Table 5) because home BP is usually lower than the clinic BP. Home BP has higher reproducibility and is more related to TOD (especially LVH) than clinic BP [16]. Furthermore, home BP is known to better predict potential cardiovascular disease and death compared to clinic BP [61]. A recent body of evidence supported that HBPM improves compliance to medication and BP control. Remote monitoring using a smartphone application is expected to have more benefits for monitoring home BP.

**Table 5:** 2018 ESC/ESH Guidelines for the management of arterial hypertension

Variable	SBP (mmHg)		DBP (mmHg)
Office BP	≥ 140	and/or	≥ 90
24-Hour ambulatory BP	≥ 130	and/or	≥ 80
Home BP	≥ 135	and/or	≥ 85

*ESC/ESH* European Society of Cardiology/European Society of Hypertension, *BP* blood pressure, *DBP* diastolic BP, *SBP* systolic BP

#### ACC/AHA guidelines for the management of hypertension

ACC/AHA (American College of Cardiology/American Heart Association) published a new version of the Guideline for the Prevention, Detection, Evaluation, and Management of High Blood Pressure in Adults in 2017 and published the “Self-measured blood pressure monitoring at home: a joint policy statement from the American Heart Association and American Medical Association” in 2020 [62, 63]. The guideline published in 2017 recommends monitoring BP outside clinics (home BP, ambulatory BP) for hypertension diagnosis and management of antihypertensive medication (grade I recommendation, level of evidence A) [38, 63]. Although ABPM is generally recognized as the optimal method, HBPM is more practical for clinical practice. Since most home BPs tend to be lower than clinic BPs, the US clinical practice guidelines provide a reference table of clinic BPs corresponding to home BPs (Table 6). In addition, more specific guidelines are presented for clinicians and patients on how to accurately monitor and utilize home BP (Table 7). Home BP monitored in such a manner showed a strong correlation with cardiovascular mortality that was independent of clinic BP [64].

**Table 6:** Corresponding values of SBP/DBP for office, 24-h ambulatory, and home BP measurement of 2017 ACC/AHA blood pressure treatment guideline

BP category	Office BP (mmHg)	24-h ambulatory BP (mmHg)	Home BP (mmHg)
Normal	120/80	115/75	120/80
Stage 1 hypertension	130/80	125/75	130/80
Stage 2 hypertension	140/90	130/80	135/85
	160/100	145/90	145/90

*BP* blood pressure, *SBP* systolic BP, *DBP* diastolic BP, *ACC/AHC* American College of Cardiology/American Heart Association

**Table 7:** Recommendations of home BP measurement of 2017 ACC/AHA Blood Pressure Treatment Guideline

Category	Recommendation
Patient training should occur under medical supervision	Information about hypertension, selection of prompt equipment, acknowledgement that individual BP readings may vary substantially, and interpretation of measurement results
Device for BP measurement	Use of validated automated devices Use of auscultatory devices is not generally useful for HBPM Use of appropriate cuff size to fit the arm Verify that right/left inter-arm BP differences are insignificant. If BP differences are significant (> 10 mmHg), instruct patient to measure BP in the arm with higher BP readings
Instructions on HBPM procedures	Avoid smoking, caffeine beverages, or exercise within 30 min before BP measurement Ensure more than 5 min of quiet resting before BP measurement Sit with back straight and supported on a straight-backed chair Sit with feet flat on the floor with legs uncrossed Keep arm supported on a flat surface with the upper arm at heart level Place the cuff directly above the antecubital fossa with bend of the elbow BP measurement at least two times 1 min apart before taking antihypertensive medications in the morning and twice before supper in evening Record all readings accurately, BP should be based on an average of readings on more than two occasions for clinical decision making

*ACC/AHC* American College of Cardiology/American Heart Association, *BP* blood pressure, *HBPM* home blood pressure monitoring

In 2020, ACC/AHA published their Clinical Practice Guideline for Self-Measured Blood Pressure Monitoring At Home [62]. This includes the following main content: (1) home BP should be recognized as a necessary part of the diagnosis and treatment of hypertension as a validated method among the BP monitoring methods outside the clinic; (2) elevated home BP is associated with cardiovascular risks independent of clinic BP; and (3) the superiority of home BP in predicting cardiovascular risk compared to ambulatory BP requires further research. The ACC/AHA Clinical Practice Guideline suggests the following as indications for HBPM: (1) white coat hypertension and masked hypertension; (2) assessment of treatment response; (3) diagnosis of resistant hypertension; and (4) detection of morning hypertension. The ACC/AHA Guideline also states that self-monitored BP is more practical than ambulatory BP for patients on antihypertensive medication. The guideline also addressed that home BP is more cost-effective than clinic BP monitoring in patients with high clinic BP. Recently, in the United States, the purchase of a home BP manometer is covered by medical insurance to encourage the effective use of HBPM. In addition, there have been initiatives to support consultation fees for HBPM training and interpretation, inspired by the clinical practice committee.

#### KSH guidelines for the management of hypertension

In Korea, the Guidelines for Blood Pressure Monitoring was published primarily by the working group for BP monitoring of KSH and home BP was covered in the "Self-Measurement of Blood Pressure" section [65]. Thereafter, new content was presented along with the existing content whenever new KSH Guidelines for the Management of Hypertension were published. In the Guidelines for the Management of Hypertension published by the KSH in 2018, "home BP measurement" was included as part of BP monitoring [62].

The main recommendations include the following: (1) HBPM is recommended for the diagnosis and assessment of prognosis of hypertension, white coat hypertension, and masked hypertension (grade I recommendation, level of evidence A); and (2) for accurate home BP measurements, accurate measurement method should be educated to all patients (grade I recommendation, level of evidence C). Home BP is more useful in predicting the incidence of cardio-cerebrovascular disease than clinic BP in patients with hypertension. Since its usefulness is high in terms of the medical economy, the importance of home BP in the diagnosis and the management of hypertension, is emphasized in the hypertension guidelines [66]. And HBPM helps evaluate masked hypertension, white coat hypertension and resistant hypertension. In addition, since home BP can accurately evaluate the BP control status of patients receiving the antihypertensive drug, it produces positive results in improving patient compliance and BP control rate [67, 68]. Therefore, it is important to monitor accurately using proper methodology, and the acurrate HBPM methodology can be found in the clinical practice guidelines.

In the Guidelines for the Management of Hypertension published by the KSH in 2018, hypertension using HBPM is defined as ≥ 135/85 mmHg and “morning hypertension” is defined as cases in which BP measured in the morning is ≥ 135/85 mmHg and is higher than the BP before sleep. However, a home BP level that corresponding to normal BP and a target BP for treatment could not be presented.

#### JSH guidelines for the management of hypertension

In 2012, the JSH published "Self-Monitoring of BP at Home (2nd edition)," a guideline for HBPM, with Professor Imai as the lead author [26]. Many sections, including the diagnostic criteria for home BP described in the guidelines, were based on the contents in the 2009 JSH Guidelines for the Management of Hypertension [69].

In this guideline, the diagnostic criteria for hypertension using home BP were defined as ≥ 135/85 mmHg, which was mostly selected based on previous cross-sectional studies and guidelines [70]. According to the Ohasama study, which was the basis for diagnostic criteria, the relative risk (RR) of cardiovascular mortality was minimal when home BP was 120–127/72–76 mmHg, and the RR increased significantly when home BP reached 138/83 mmHg. In addition, based on this study, a normal value for home BP of < 125/80 mmHg and a high normal range of 125/80–134/84 mmHg were suggested [71].

In 2008, the AHA published a statement with the American Society of Hypertension. Based on previous studies, target home BPs were set at < 135/85 mmHg for the general population, < 130/80 mmHg for high-risk patients and < 125/75 mmHg for diabetic patients. In addition, based on the results of a previous meta-analysis, normal home BP (optimal home BP) was reported to be < 120/80 mmHg [72]. Based on previous publications and intervention studies using HBPM, the 2012 home BP guidelines suggested target BPs for clinic and home BP by comorbid diseases. The target home BPs for young/middle-aged general hypertensive patients were < 130/85 mmHg and < 125/85 mmHg, respectively. Furthermore, target clinic and home BPs for elderly hypertensive patients were < 140/90 mmHg and < 135/85 mmHg, cerebrovascular disorder were < 140/90 mmHg and < 135/85 mmHg, and diabetic patinets and patintients with CKD or myocardial infarction (MI) were < 130/80 mmHg and < 125/75 mmHg, respectively. Since then, the home BP guidelines have not been revised, and the contents related to HBPM have been included in the JSH Guidelines for the Management of Hypertension.

The Guidelines for the Management of Hypertension published by JSH in 2019 also included HBPM as “measurement and clinical evaluation of BP” [72]. In addition, it was highlighted that home BP is critical in establishing a diagnosis of and treatment strategy for white coat hypertension or masked hypertension, morning hypertension and resistant hypertension. The benefits of and correct monitoring methodology for home BP monitor were described. It was emphasized that the mean of morning and evening values measured for at least 5–7 days are essential for the diagnosis and assessment of hypertension, normal BP, and antihypertensive drug effects. The criteria for diagnosing hypertension was defined as mean morning or evening home BP ≥ 135/85 mmHg, as in previous studies and hypertension guidelines. Additionally, these guidelines define normal home BP as the mean morning and evening BP < 115/75 mmHg. Home BPs corresponding to clinic BP criteria are presented in Table 8.

**Table 8:** 2019 Japanese Society of Hypertension classification of BP levels

Category	Office BP (mmHg)			Home BP (mmHg)		
	SBP		DBP	SBP		DBP
Normal BP	< 120	and	< 80	< 115	and	< 75
High normal BP	120–129	and	< 80	115–124	and	< 75
Elevated BP	130–139	and/or	80–89	125–134	and/or	75–84
Grade I HTN	140–159	and/or	90–99	135–144	and/or	85–89
Grade II HTN	160–179	and/or	100–109	145–159	and/or	90–99
Grade III HTN	≥ 180	and/or	≥ 110	≥ 160	and/or	≥ 100
(Isolated) Systolic HTN	≥ 140	and	< 90	≥ 135	and	< 85

*BP* blood pressure, *SBP* systolic BP, *DBP* diastolic BP, *HTN* hypertension

The 2019 JSH Guidelines strongly recommend antihypertensive treatment based on home BP rather than office BP (grade I recommendation, level of evidence A), as home BP is more reproducible and correlates with cardiovascular disease. Furthermore, home BP monitoring is useful for diagnosing white coat hypertension and masked hypertension. It is also actively recommended for patients undergoing antihypertensive treatment as it helps to evaluate hypotension during antihypertensive treatment and improves patient adherence to antihypertenive medication.

Intervention studies using home BP have been performed previously, but they did not provide sufficient evidence. Based on the Ohasama and HOMED-BP studies, it was proposed that the target home BP should be set 5 mmHg lower than that for clinic BP for both systolic and diastolic pressures [73–75]. Therefore, the target level of clinic BPs and the corresponding home BPs by comorbid disease are described in Table 9.

**Table 9:** 2019 Japanese Society of Hypertension, target BP by disease

Category	Office BP (mmHg)	Home BP (mmHg)
Adult patients < 75 years old	< 130/80	< 125/75
Cerebrovascular disease (without bilateral carotid artery obstruction or occlusion of cerebral artery)		
Coronary artery disease		
Chronic kidney disease (proteinuria +)		
Diabetes mellitus		
Presence of antithrombotic medications		
Elderly patients ≥ 75 years old	< 140/90	< 135/85
Cerebrovascular disease (with bilateral carotid artery obstruction or occlusion of cerebral artery, or unevaluated)		
Chronic kidney disease (proteinuria –)		

*BP* blood pressure

### Limitations of home blood pressure monitoring

HBPM using an automated device enables patients with hypertension to obtain more BP values by monitoring BP at home without limitations on time while avoiding the whitecoat effect. In addition, there are several advantages for hypertension treatment, such as enhanced compliance to antihypertensive drugs, since the patients can directly check the change of BP with medication or lifestyle modification. In particular, the use of home BP is gradually increasing as the number of validated BP manometers has increased in recent years, and the devices have become more popular as the price of manometers has gone down.

However, there are still limitations with HBPM. If these limitations are not addressed, it may cause unnecessary concerns to patients and delay appropriate actions. For this reason, health care providers should be fully aware of this and provide education to patients. There are several limitations of HBPM that may become clinically problematic.

### Arrhythmias

**Fig. 7:**
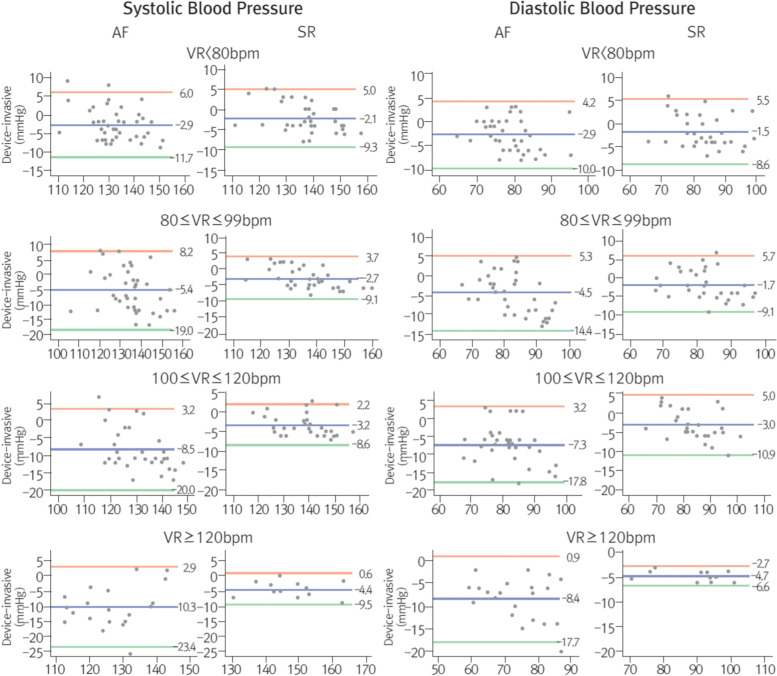
Measurement error between blood pressure by heart rate in atrial fibrillation and normal sinus rhythm. AF, atrial fibrillation; BPM, beats per minute; SR, sinus rhythm; VR, ventricular rate. Reprinted from Pagonas et al. [76] with permission of Wolters Kluwer Health

Measurement of home BP using oscillometric method may be inaccurate in the presence of severe arrhythmia. Accordingly, devices capable of detecting arrhythmia such as atrial fibrillation have been developed, which may help the diagnosis of paroxysmal atrial fibrillation, which is challenging to diagnose. In the case of arrhythmias, errors in the BP measurement using automated devices are inevitable. According to a study by Pagonas et al. [76] in 2013, there was no significant difference between BP measured in the presence of atrial fibrillation and normal rhythm when using Omron M5/R5 Professional devices (Omron, Kyoto, Japan) (Fig. 7). Therefore, even if there is atrial fibrillation, the measurement of BP is not an issue if the measurement is repeated three times [76].

Xie et al. [77] investigated whether BP measurement errors were due to rapid heart rate in patients with atrial fibrillation and reported that in patients with persistent rapid heart rate due to atrial fibrillation, the monitored oscillometric BP was significantly lower even with repeat measurements. Therefore, it should be noted that there can be errors with BP monitoring in patients whose heart rate is not controlled due to arrhythmia.

### Pregnant women

Gestational BP monitoring is critical to the diagnosis of gestational hypertension. BP during pregnancy directly impacts the mother's health and fetal development, and BP must be accurately monitored using a proper method to determine whether to treat hypertension and maintain proper target BP. In other words, receiving unnecessary treatment due to incorrectly detected high BP resulting from a white coat effect and missing the optimal treatment period by depending on clinic BP and not recognizing masked hypertension may cause problems, including progression of eclampsia. Therefore, BP must be monitored accurately through HBPM or ABPM. However, due to the increase in blood volume and decrease in peripheral vascular resistance, the pulse pressure widens during pregnancy. Thus, oscillometric HBPM may be inaccurate. It should also be considered that there is not much research-validated using conventional BP manometers in pregnant women. Even though a BP manometer is validated, it cannot be considered suitable during pregnancy. According to a meta-analysis done by Bello et al. [78] in 2018, the Microlife Watch BP Home and Omron MIT BP manometers are the only two home BP monitors validated in pregnant women that do not have any methodological errors or violations of regulations.

### Nocturnal hypertension

**Fig. 8:**
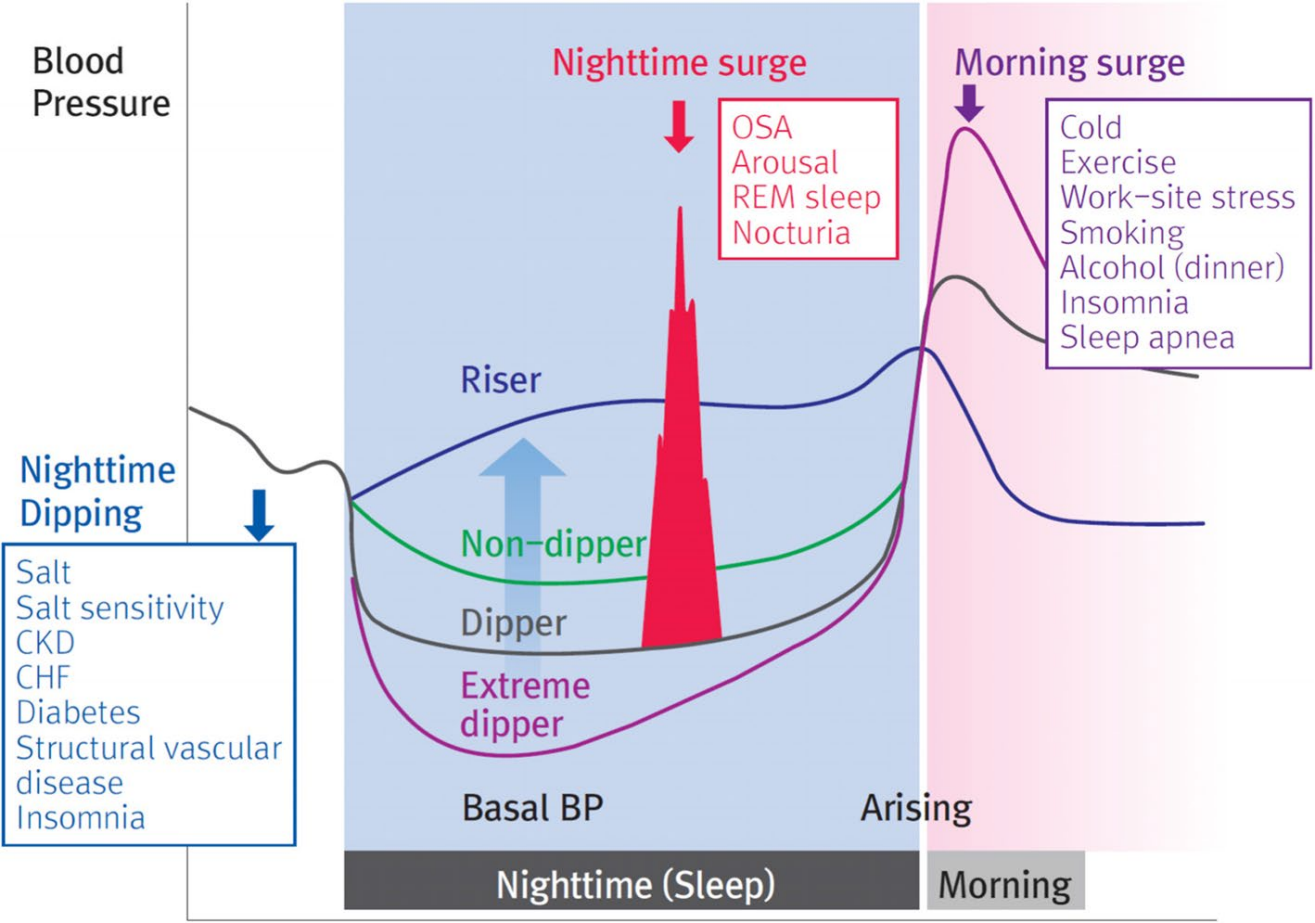
Factors related with nocturnal hypertension. BP, blood pressure; CKD, chronic kidney disease; CHF, congestive heart failure; OSA, obstructive sleep apnea; REM, rapid eye movement. Reprinted from Kario et al. [79] with permission of Wolters Kluwer Health

HBPM may be superior to ABPM in terms of convenience and capability for repeated measurement; however, its limitation is the difficulty in BP monitoring during sleep. Importantly, this limitation lies in that assessment of nocturnal hypertension due to sleep apnea, nighttime dipping, and morning surge is not feasible (Fig. 8).

The 2019 International Expert Group of Nocturnal Home Blood Pressure has reported that home nocturnal BP monitoring using automated devices, including the Microlife Watch BPN (Microlife, Taipei, Taiwan) and Omron HEM7252G-HP, showed a comparable level of accuracy with ambulatory nocturnal BP monitoring [18]. However, questions on the clinical significance of nocturnal BP monitored at home, and the appropriate nocturnal BP monitoring interval should be addressed by further research to enhance the use of home nocturnal BP monitoring using these devices.

## Self-blood pressure monitoring using mobile devices

Accurate BP monitoring is the most critical aspect of hypertension management. Accurate home BP monitoring better reflects prognosis than clinic BP and can enhance medication compliance and treatment response. Smartwatches and smartphones are essential personal mobile devices. It is estimated that at least 5 billion people possess mobile devices and more than half of these people have smartphones. According to a report by the Korea Information Research and Development Institute in 2018, 37.8% of Koreans ≥ 70 years old had smartphones, tenfold the percentage in 2013, which was 3.6% [80], from 19% to 80.3% among those in their 60 s, 51.3% to 95.5% among those in their 50 s, 81.3% to 98.4% among those in their 40 s, and 94.2% to 98.7% among those in their 30 s. Meanwhile, the percentage of people possessing smartphones was among the highest in the world, exceeding that in developed countries (95%), including Israel (88%) and the Netherlands (87%). Similarly, various wearable devices that monitor BP using mobile device sensors and signal analysis are being developed and improving more rapidly in recent years [81]. In particular, the Galaxy Watch of Samsung Electronics (Suwon, Korea) has met the requirements for medical devices for the first time globally. Therefore, this section describes the current milestones of self-measured BP monitoring using mobile devices, the monitoring method, and the limitations of these devices.

### Current milestones of BP monitoring using mobile devices

Although prior research has reported that the accuracy of BP monitoring using smartphones is 95– 100%, actual measurements are presented as a range rather than specific values as a result may fluctuate depending on the measurement method [82]. Even though there were difficulties in developing a mobile device/algorithm that meets medical device standards (AAMI), the data has improved remarkably with the application of the ubiquitous model and linear polynomial equation [83, 84]. A study using the iPhone’s BP monitoring via the oscillometric finger-pressing method found an error range of –4.0 mmHg for systolic pressure and –9.4 mmHg for diastolic pressure, which is within the acceptable error range for existing finger BP monitors approved as medical devices and the AAMI criteria of 5 ± 8 mmHg [85, 86].

BP monitoring using a smartwatch has two challenges. First, the accuracy of photoplethysmographic sensors is not yet validated. If there is a near-infrared light source around the site of BP monitoring, spot monitoring can be less accurate. Second, self-monitoring is prone to error despite proper training. The error can be even more prominent with unstable resting posture.

### Guideline on BP monitoring using smartwatches

BP obtained through a conventional monitoring device should be entered periodically into the BP monitoring app in the smartphone to monitor BP with a smartwatch. Paradoxically, monitoring BP with a mobile device has collateral benefits. BP should be monitored periodically with a conventional BP manometer and the mobile device and eventually check the BP more accurately. It is recommended to perform the BP measurement three times, 2 min apart for the calibration, and it must be done again if switching the smartwatch to the other wrist.

The core problem in the adjustment process is the difference in BPs between the left and right arms. It has been reported in many epidemiological studies that the gap between the systolic/diastolic pressures of the two arms is approximately 3.3/2.0 mmHg, respectively. Also, the BP difference between the two arms widens as BP increases. Therefore, if the calibration is performed using BP values measured in the opposite arm, it can cause an error of at least 3 mmHg, which cannot be addressed by any internal calibration mechanism.

Moreover, the clinical studies on smartwatches show that the patient can measure BP on the opposite arm with a general BP manometer while measuring BP with a smartwatch, thus compensating for the difference between the two arms and enabling a simultaneous BP measurement in both arms. However, in real life, BP cannot be measured in both arms without assistance, so the benefit of measuring BP in one arm with a smartwatch while measuring in the other arm with a general BP manometer is not practically obtainable. Therefore, a position paper from the KSH, smartwatch-based cuffless BP measurement recommends calibration by obtaining a BP measurement signal with a smartwatch and then measuring BP in the same arm with a general BP manometer [87]. Also, when the BP is measured with a general BP manometer, as the compression and release of the vessels of the upper arm by the cuff may affect the BP waveform, it is recommended to measure with the smartwatch first with a general BP manometer. When adjusting, it is important to repeat these steps three times, 1–2 min apart. The correct method for measuring BP with a smartwatch is summarized in Table 10.

**Table 10:** BP monitoring using smart watches

Step	Process
Step 1. Proper monitoring posture and adjustment	1. Sit comfortably in a chair with a backrest and rest for 3–5 min. Make sure your arms and wrists are not too dry or wet with lotion
	2. Avoid coffee, vigorous physical activity, and smoking for at least 30 min before monitoring
	3. Make sure to urinate before monitoring
	4. Refrain from talking during monitoring
	5. Sit comfortably with your back on a chair with a backrest and your feet on the floor without folding your legs. With your arms slightly bent, place them on a desk at the height of your heart. During monitoring, breathe comfortably and do not hold your breath or breathe rapidly
	6. For adjustment, wear the general BP manometer on the upper arm of the arm where the smart watch is worn
Step 2. Adjustment	1. Fasten the smart watch strap enough to ensure the watch contacts the wrist without being too tight
	2. Launch the BP monitoring app in the smart watch and wait until it receives the signal
	3. Monitor the BP using the general BP manometer on the upper arm
	4. Enter the monitored value in the BP monitoring app. Repeat this 3–5 times for adjustment
Step 3. Monitoring	Maintain the right posture explained in Step 1 and continue monitoring the BP
Step 4. Periodic re- adjustment	Re-adjust periodically to maintain correctness The value must be re-adjusted if the arm wearing the watch has changed or if another person is wearing the smart watch

*BP* blood pressure

Devices certified as medical devices are not recommended for patients with a systolic BP that is considered very high ≥ 160 mmHg or very low ≤ 80 mmHg since their accuracy is not yet validated. They are also not recommended for patients with aortic insufficiency, atrial fibrillation with high heart rate variability, peripheral vascular disease with weak blood flow, glycosuria, cardiomyopathy, end-stage renal failure, hand tremor, or blood coagulation disorder. In addition, they are not recommended for patients taking antiplatelet/anticoagulant drugs or states with hormone fluctuation, such as pregnancy, since their vascular characteristics differ from the normal range.

Therefore, accurate self-measured BP monitoring using a smartwatch is achieved only when a standardized procedure is followed. However, it is not recommended for patients with systolic BP considered very high ≥ 160 mmHg or very low ≤ 80 mmHg since it is not well established in these populations.

### Prospects of self-measured BP monitoring using mobile devices

So far, BP monitoring using mobile devices has been primarily effective in raising awareness of the importance of BP management among the general public and the early diagnosis of hypertension rather than monitoring patients with hypertension. Implementation in the geriatric population may be limited as they may find it difficult to adjust the device using a general BP manometer periodically. However, its significance lies in raising awareness of hypertension in tech-savvy adults in their 30 s to 40 s, triggering early hypertension management.

On the other hand, periodic BP monitoring (using general manometers) with mobile devices can facilitate routine BP monitoring in patients with hypertension. However, we cannot exclude the risk that patients may self-adjust medication based on a BP result inappropriately measured in an unrelaxed state. In addition, various studies have reported that mobile devices' BP monitoring results indicated significant differences over 24-h periods, between days of the week, and even from season to season [87]. Most importantly, as there are remaining concerns that inappropriately measured BP may cause unnecessary stress to mental health, incorrect diagnoses, and increases in medical expenses, a cost-effectiveness analysis of BP monitoring using mobile devices is deemed necessary.

Lastly, BP monitoring using a mobile device can open up a new field for assessing the dynamic changes in BP [88]. Only static evaluation of BP after a period of sufficient rest has been considered accurate. However, many studies have proven BP variability to be an independent and powerful indicator of future cardiovascular events. Suppose BP changes due to daily life and physical/emotional stress can be identified through mobile device-based BP monitoring. In that case, it is speculated that a study on the association between BP changes and cardiovascular disease in these situations will be feasible.

## Conclusions

This position paper is an official opinion of Korean Society of Hypertension Home Blood Pressure Forum. HBPM has advantages, including effectively diagnoses stress-induced transient BP elevations (i.e., white coat hypertension), insufficient BP control throughout the day (i.e., masked hypertension), and even BP variability. Additionally, HBPM may increase self-awareness of BP, increasing the compliance of treatment. Cumulative evidence has reported better improved predictive values of HBPM in cardiovascular morbidity and mortality than office BP monitoring.

## Data Availability

Not applicable.

## Abbreviations

ACC/AHA: American College of Cardiology/American Heart Association
ABPM: Ambulatory blood pressure monitoring
AAMI: Association for the Advancement of Medical Instrumentation
BP: Blood pressure
BHS: British Hypertension Society
CKD: Chronic kidney disease
CI: Confidence interval
ESC: European Society of Cardiology
ESH: European Society of Hypertension
ESH-IP: European Society of Cardiology-International Protocol
HR: Hazard ratio
HBPM: Home blood pressure monitoring
IMT: Intima-media thickness
ISO: Organization for Standardization
J-HOP: Japan Morning Surge-Home Blood Pressure
JSH: Japanese Society of Hypertension
KSH: Korean Society of Hypertension
LV: Left ventricular
LVH: Left ventricular hypertrophy
MUCH: Masked uncontrolled hypertension
PAMELA: Pressioni Arteriose Monitorate E Loro Associazioni
RR: Relative risk
TOD: Target organ damage
WUCH: White coat uncontrolled hypertension
